# Propyl-5-hydroxy-3-methyl-1-phenyl-1*H*-pyrazole-4-carbodithioate (HMPC): a new bacteriostatic agent against methicillin—resistant *Staphylococcus aureus*

**DOI:** 10.1038/s41598-018-25571-w

**Published:** 2018-05-04

**Authors:** Tatiana Johnston, Daria Van Tyne, Roy F. Chen, Nicolas L. Fawzi, Bumsup Kwon, Michael J. Kelso, Michael S. Gilmore, Eleftherios Mylonakis

**Affiliations:** 1 Department of Infectious Disease, Rhode Island Hospital, Alpert Medical School of Brown University, Providence, Rhode Island USA; 2 Department of Ophthalmology, Harvard Medical School, Massachusetts Eye and Ear Infirmary, Boston, Massachusetts USA; 3 Department of Microbiology and Immunobiology, Harvard Medical School, Boston, Massachusetts, USA; 4 Department of Molecular Pharmacology, Physiology, and Biotechnology, Brown University, Providence, Rhode Island USA; 5 Department of Neurology, Rhode Island Hospital, Warren Alpert Medical School of Brown University, Providence, RI 02903 USA; 6 School of Chemistry and Illawarra Health and Medical Research Institute, University of Wollongong, Wollongong, NSW 2522 Australia

## Abstract

The emergence of *Staphylococcus aureus* strains resistant to ‘last resort’ antibiotics compels the development of new antimicrobials against this important human pathogen. We found that propyl 5-hydroxy-3-methyl-1-phenyl-1*H*-pyrazole-4-carbodithioate (HMPC) shows bacteriostatic activity against *S. aureus* (MIC = 4 μg/ml) and rescues *Caenorhabditis elegans* from *S. aureus* infection. Whole-genome sequencing of *S. aureus* mutants resistant to the compound, along with screening of a *S. aureus* promoter-*lux* reporter array, were used to explore possible mechanisms of action. All mutants resistant to HMPC acquired missense mutations at distinct codon positions in the global transcriptional regulator *mgrA*, followed by secondary mutations in the phosphatidylglycerol lysyltransferase *fmtC/mprF*. The *S. aureus* promoter-*lux* array treated with HMPC displayed a luminescence profile that was unique but showed similarity to DNA-damaging agents and/or DNA replication inhibitors. Overall, HMPC is a new anti-staphylococcal compound that appears to act via an unknown mechanism linked to the global transcriptional regulator MgrA.

## Introduction

*Staphylococcus aureus* is a highly adaptive human pathogen that is responsible for a variety of diseases ranging from soft tissue infections to bacteremia, endocarditis, pneumonia and osteomyelitis^1–3^. According to a report from the US Centers for Diseases Control, the resistance of *S. aureus* to methicillin and related beta-lactams, as well as to cephalosporins poses a large concern for public health. The CDC reported that methicillin-resistant *S. aureus* (MRSA) alone was responsible for 80,461 invasive infections and 11,285 related deaths in 2011^4^. The organism possesses a remarkable ability to survive by adapting to changing environmental conditions and by mobilizing complex defense mechanisms in response to exogenous stressors^5^. The overuse of antibiotics in medicine and agriculture, together with the use of compounds that target essential cellular functions, such as DNA replication, RNA transcription and protein and cell wall synthesis, have put selective pressure on bacteria leading to multidrug-resistant strains that are both challenging and expensive to treat^6–8^. The loss of potency of antibiotics to treat *S. aureus* infections poses a serious threat to public health, as evidenced by the emergence of methicillin-resistant *S. aureus* (MRSA) and vancomycin-resistant *S. aureus* (VRSA) strains^9–12^. The resistance of *S. aureus* to ‘last resort’ antibiotics (vancomycin, and more recently daptomycin and linezolid) has placed this bacterium on the World Health Organization’s list of high priority antibiotic-resistant bacteria for which there is an urgent need to develop effective new treatments^13^.

In this study, we describe the antimicrobial properties of propyl 5-hydroxy-3-methyl-1-phenyl-1*H*-pyrazole-4-carbodithioate (HMPC) as a new agent against *S. aureus* and suggest that it likely acts via a novel mechanism of action.

## Results

### Antimicrobial activity

**Figure 1:**
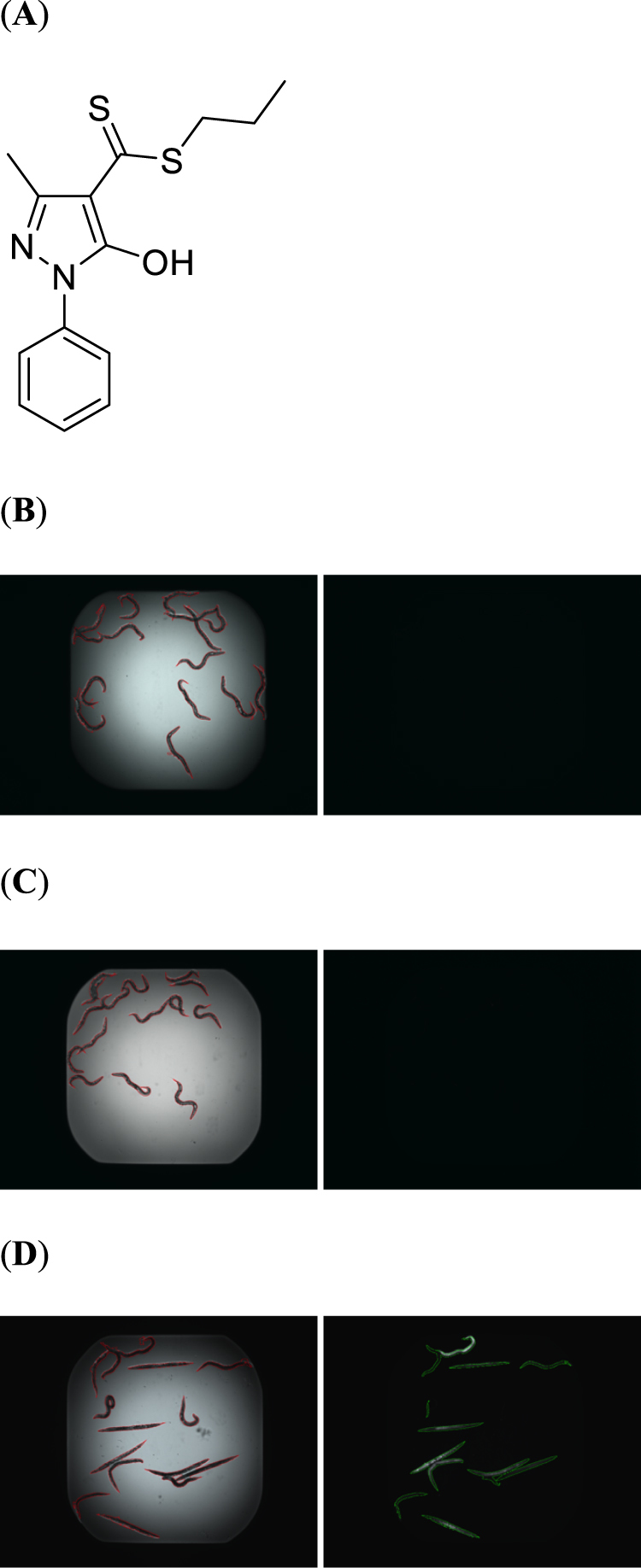
HMPC rescues *C. elegans* from MRSA infection. (**A**) Chemical structure of propyl 5-hydroxy-3-methyl-1-phenyl-1*H*-pyrazole-4-carbodithioate (HMPC). (**B–D**) Results of compound screening for *C. elegans* viability following *S. aureus* MW2 infection. A 384-well assay plate was co-inoculated with *C. elegans*, *S. aureus* MW2 and either 7 μg/ml HMPC (**B)**; 100% survival), 10 μg/ml vancomycin (**C**) positive control; 100% survival), or 1% DMSO vehicle (**D**) negative control; 40% survival). Representative Sytox Orange-stained (right) and bright field (left) images from assay wells are shown. Only dead worms take up Sytox Orange and fluoresce. The images were first captured with an ImageExpress and further processed with CellProfiler to normalize the fluorescent or bright field intensity and to define the area of each worm.

Screening of the Maybridge 5 Library (Maybridge, Thermo Fisher Scientific) identified HMPC as a compound with direct inhibitory activity against *S. aureus* MW2 (MIC = 4 μg/ml, Fig. 1A)^14^ and the ability to rescue *C. elegans* from *S. aureus* MW2 infection^15–18^, with the fraction of *C. elegans* survival ranging between 93–100% after 5 days of co-incubation (Fig. 1B–D). HMPC appeared to not affect the viability of *C. elegans* at the screening concentration of 7 μg/ml. The compound was found to be equipotent across a panel of recent clinical isolates of methicillin-resistant *S. aureus* from our laboratory collection, where all tested isolates had MICs = 4 μg/ml (Table 1).

**Table 1:** Activity of HMPC against methicillin-resistant *S. aureus* (MRSA) clinical isolates.

*S. aureus* strain	HMPC MIC (µg/ml)	Oxacillin MIC (µg/ml)^a^	Vancomycin MIC (µg/ml)^b^
BFSA25	4	32	2
BFSA30	4	32	1
BFSA31	4	64	2
BFSA32	4	64	2
BFSA33	4	64	2
BFSA48	4	>64	1

^a,b^MIC values for oxacillin and vancomycin are shown for comparison.

**Figure 2:**
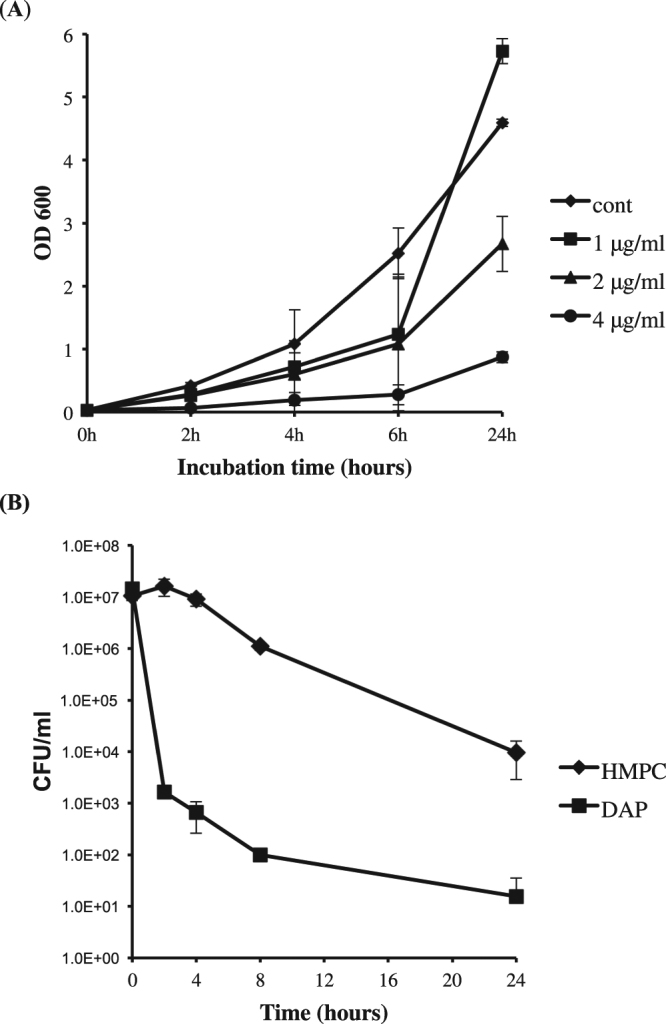
(**A**) *S. aureus* MW2 growth inhibition in the presence of 1–4 μg/ml HMPC. Bacterial culture aliquots were periodically taken during log-phase growth to measure the optical density at 600 nm (OD_600_). Data show the mean +/− standard deviation of triplicate values, and significant differences from the control were observed for all concentrations tested (p < 0.05 by two-tailed t-test). (**B**) Killing assay with HMPC or daptomycin (DAP). Log-phase cultures were treated with 16 μg/ml of HMPC (4xMIC) or 10 μg/ml DAP and were incubated at 37 °C with shaking at 220 rpm. Bacterial survival was monitored after 2, 4, 8, and 24 hours by dilutional plating and enumeration of CFU/ml at each time point. The differences between HMPC and DAP are significant at all time points except T0 (p < 0.05 by two-tailed t-test).

The spectrum of activity of HMPC was evaluated by determining its MIC against a panel of ESKAPE (*Enterococcus faecium*, *Staphylococcus aureus*, *Klebsiella pneumoniae*, *Acinetobacter baumannii*, *Pseudomonas aeruginosa*, and *Enterobacter* species) pathogens^19,20^. In addition to *S. aureus*, the compound was also found to be active against *Enterococcus faecium* (MIC = 16 μg/ml), but showed no activity against the gram-negative strains tested (Table 2). Growth curves of the *S. aureus* MW2 strain in the presence of increasing concentrations of HMPC showed that the compound was able to inhibit growth in a concentration-dependent manner over the range 1–4 μg/ml (Fig. 2A). We also performed a time-kill assay using a high concentration of HMPC (16 μg/ml, 4xMIC) and compared the killing kinetics of HMPC to the rapidly bacteriocidal antibiotic daptomycin (Fig. 2B). We found that HMPC did not cause a reduction in bacterial viability until eight hours post-treatment, in contrast to the rapidly bacteriocidal nature of treatment with daptomycin.

**Table 2:** Activity of HMPC against a panel of ESKAPE pathogens. Vancomycin and gentamicin were included for comparison purposes.

Pathogen	HMPC, MIC (μg/ml)	Vancomycin, MIC (μg/ml)	Gentamicin, MIC (μg/ml)
*Enterococcus faecium* E007	16	1–2	
*Staphylococcus aureus* MW2	4	1	
*Klebsiella pneumoniae* WGLW2	>64		0.5
*Acinetobacter baumannii* ATCC 17978	>64		1
*Pseudomonas aeruginosa* PA14	>64		2–4
*Enterobacter aerogenes* Hormaeche and Edwards ATCC 13048	>64		1

### Screening of a *S. aureus* promoter-lux clone array

**Figure 3:**
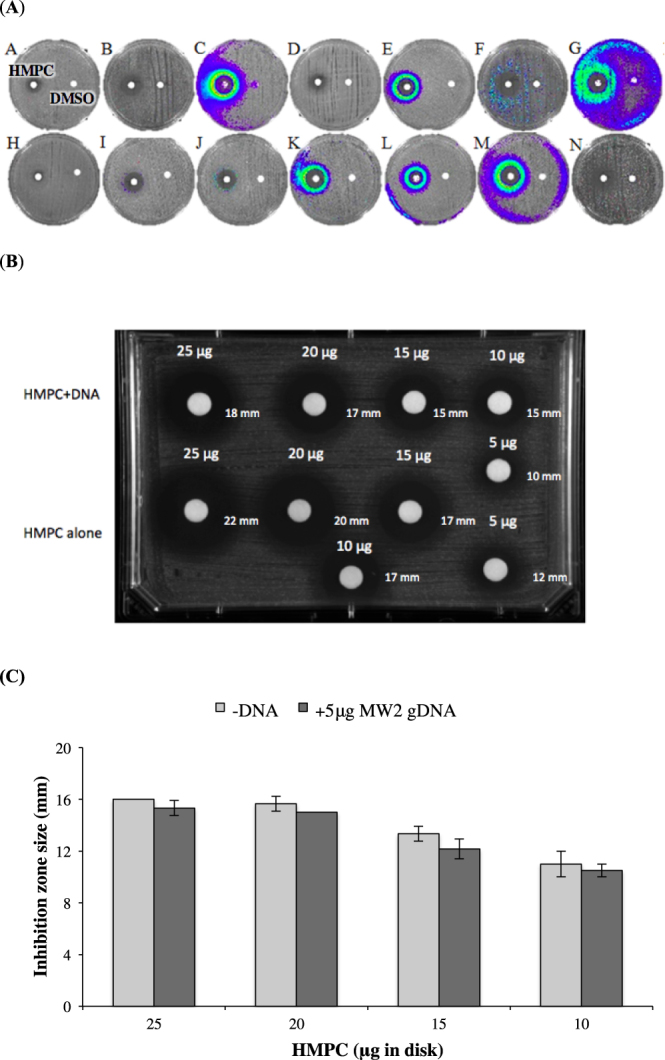
Response of the *S. aureus* promoter-*lux* array to HMPC treatment. (**A**) Results of promoter-*lux* array screening. The name of each reporter clone is shown in the upper left corner of each picture. A clear zone around the disc indicates inhibition of growth in the presence of higher concentrations of HMPC. At the periphery of the inhibition zone, where sub-MIC levels of HMPC are present, changes in luminescence indicate altered transcription. The disc on the left is HMPC and on the right of each panel is a DMSO (vehicle) control. (**B**) Zones of *S. aureus* growth inhibition produced by HMPC in the presence or absence of calf thymus DNA. Decreasing quantities of HMPC (25–5 μg) were incubated with either 5 μg calf thymus DNA or TE buffer (control) before being spotted onto discs and placed onto agar plates containing *S. aureus* MW2 bacterial lawns. (**C**) Disc diffusion assay with varying concentrations of HMPC in the absence or presence of *S. aureus* genomic DNA. HMPC was incubated without or with 5 μg of *S. aureus* genomic DNA and was then applied to a filter paper disc and placed onto a TSA plate containing *S. aureus* MW2 bacteria in a soft agar overlay. After overnight incubation at 37 °C, zones of inhibition were measured with a ruler. Bars show mean values +/− standard deviation of three independent assays, and no differences are statistically significant.

Antibiotic-activated promoter-*lux* clone arrays in *S. aureus* provide characteristic transcriptional activation light signatures that can be used to discriminate between different antibiotic classes based on their mechanism(s) of action^21^. We screened a promoter-*lux* array to investigate whether HMPC induces transcriptional changes in *S. aureus* similar to those of known classes of antibiotics. HMPC was found to activate promoter-*lux* clones C, E, F, G, K, L and M (Fig. 3A), which appears to be a unique profile but shows some overlap with DNA-damaging agents and/or DNA replication inhibitors^21,22^.

To test whether HMPC interacts directly with DNA, we first looked for evidence of binding *in vitro* by examining the UV visible spectra of HMPC in the absence or presence of excess calf thymus DNA (Fig. S1). No DNA hyperchroism or hypochromism was observed, nor were any shifts in positions of absorption bands in the region 290–360 nm, suggesting that HMPC does not interact with eukaryotic DNA. We confirmed this observation by measuring the zones of *S. aureus* growth inhibition produced by different concentrations of the compound in the presence or absence of excess calf thymus DNA (Fig. 3B). The insignificant 3 mm inhibition zone decrease was observed in the presence of calf thymus DNA, suggesting that HMPC does not tightly bind to DNA. To test whether the compound binds to *S. aureus* DNA, we conducted disc diffusion assays using varying concentrations of HMPC with and without *S. aureus* MW2 genomic DNA (Fig. 3C). A consistent decrease in the size of the inhibition zone when DNA is included was observed across HMPC concentrations, but the effect was very small and was not statistically significant. Finally, we conducted an electrophoretic mobility shift assay (EMSA) on *S. aureus* DNA incubated with HMPC or DMSO solvent alone, which did not show evidence of direct binding (Fig. S2). The unique promoter-*lux* profile produced by HMPC therefore supports it being the prototypical member of a new antibiotic class that acts via a novel mechanism. Mesak *et al*. postulated that new compounds could potentially produce new promoter-*lux* profiles^21^ which may apply to HMPC. Additionally, the *S. aureus* RN4220 strain used to created the promoter-*lux* array possesses mutations that affect the virulence and fitness of the strain, which could influence the screening results of novel compounds^23^.

### Isolation and whole-genome sequencing of HMPC-resistant mutants

Single-step resistance selection, whereby *S. aureus* MW2 bacteria were plated onto 2x and 4x MIC, produced no HMPC-resistant bacteria (data not shown); sequential passaging in liquid medium with increasing concentrations of HMPC, however, allowed for the selection of low-level resistant mutants^24^. Mutant populations with increased MICs of either 8 μg/ml or 16 μg/ml HMPC were confirmed following several passages in non-selective medium. Three independent resistance selections were conducted and whole genome sequences were obtained from controls and corresponding mutants with MICs of 8 μg/ml or 16 μg/ml (a total of 9 populations sequenced; Table 3). Analysis of high-frequency mutations identified two genes that were repeatedly and independently mutated across multiple selections: the transcriptional regulator *mgrA (MW0648)* and the phosphatidylglycerol lysyltransferase *fmtC/mprF (MW1247)*. Further analysis of low-frequency mutants in all selected strains revealed additional mutations in both *mgrA* and *fmtC* (Table 3). One selected strain also acquired a mutation in a hypothetical protein (*MW0910*) containing a YdiL-like membrane protease domain.

**Table 3:** Mutations identified in HMPC-resistant *S. aureus* mutants selected *in vitro*.

Selection	MIC (μg/ml)	MW2 pos (bp)	Type^a^	Length (bp)	Gene ID	Frequency (%)	Protein change^b^
1	8	731157	MNV	17	MgrA	46.8	G108fs
		731244	SNV	1	MgrA	51.9	R84H
2	8	731332	SNV	1	MgrA	100	V55F
3	8	731158	Deletion	12	MgrA	52.9	L109del4
		731283	SNV	1	MgrA	28.9	P71N
		731470	SNV	1	MgrA	10.5	E9*
1	16	731244	SNV	1	MgrA	100	R84H
		1366975	Deletion	4	FmtC	93.5	I461fs
2	16	731295	SNV	1	MgrA	20.9	G67A
		731332	SNV	1	MgrA	77.4	V55F
		1366139	Deletion	7	FmtC	28	I181fs
3	16	731283	SNV	1	MgrA	100	P71N
		1005808	Insertion	1	Hyp YdiL	85	L151fs
		1366760	Deletion	684	FmtC	100	N386del228

^a^MNV = Multi-nucleotide variant; SNV = Single Nucleotide Variant.

^b^fs = frame-shift; *stop codon.

**Figure 4:**
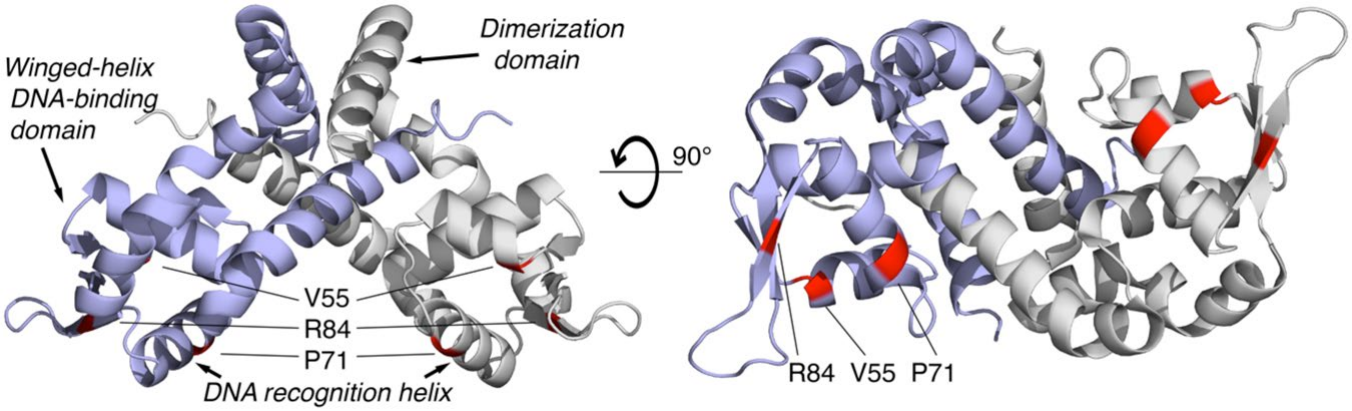
HMPC resistance-associated mutations in MgrA map to the DNA-binding domain. The MgrA dimer (PDB: 2BV6) is shown with the two equivalent subunits colored light blue and white/grey. Missense variants at Val55, Pro71, and Arg84 (shown in red) are all found in the winged-helix DNA-binding domain (left, bottom half), including in the DNA recognition helix. Image created in PyMOL with Flaticon.

We next tried to understand how the observed mutations might impact the mechanism of HMPC action and resistance. The most often mutated gene, *mgrA*, is a transcriptional regulator that controls the expression of a large number of *S. aureus* genes^25,26^. The dominant mutations observed all reside within the winged helix DNA-binding domain of the MgrA protein (Val55Phe is at the start of α-helix 3, Pro71Gln is at the start of α-helix 4 and Arg84His is in a β-strand of the wing region (PDB: 2PV6) (Fig. 4). Two resistant mutants contained a single mutation in *mgrA*, while the remaining strains possessed subpopulations of different mutants, all of which are predicted to disrupt protein function (Fig. 4 and Table 3). To test whether MgrA disruption directly impacts susceptibility to HMPC, two previously generated *S. aureus* MW2 mutants lacking a functional *mgrA* (AH3456 and AH4322) were tested for resistance to HMPC^27,28^. While no difference in MIC was observed in either mutant relative to the wild type MW2 strain AH843, both mutants were able to grow to a higher cell density at sub-MIC levels of HMPC (Fig. S3).

All three HMPC-resistant populations also developed mutations in the phosphatidylglycerol lysyltransferase *fmtC/mprF (MW1247)*, and these mutations were correlated with higher resistance to the compound (MIC = 16 μg/ml; Table 3). No *fmtC* deletion mutant exists in the MW2 strain background, but a readily available *fmtC* mutant strain was identified in the Nebraska Transposon Mutant Library (NTML, mutant NE1360, *SAUSA300_1255*). The *fmtC* transposon mutant showed a 2-fold increase in the MIC of HMPC compared to the wild type USA300 JE2 strain (MIC = 4 μg/ml; experiments were repeated twice with triplicates or duplicates). One resistant mutant also carried a mutation in *MW0910*, a hypothetical protein that contains a domain similar to the YdiL membrane protease. The corresponding NTML mutant (NE841, *SAUSA300_0932*) showed no change in MIC compared to the wild type strain (MIC = 4 μg/ml).

**Figure 5:**
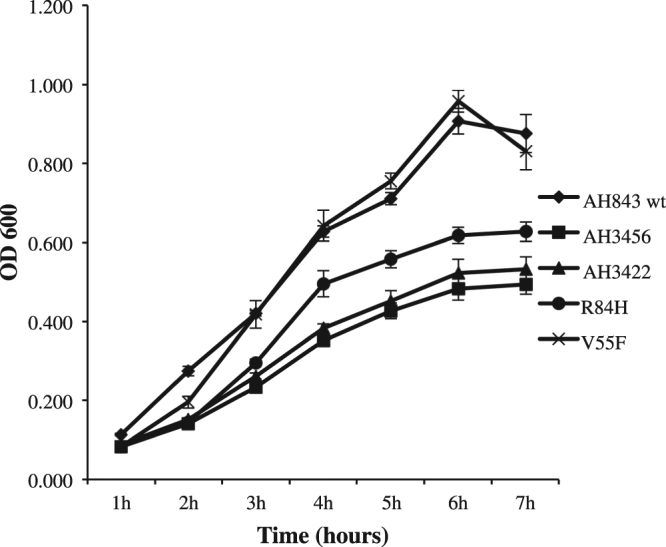
Autolysis assay for HMPC-resistant mutants containing Val55Phe and Arg84His mutations in MgrA. Bacteria were grown in a 96-well plate at 37 °C without shaking and optical density at 600 nm (OD_600_) was measured hourly. The strains AH843, AH3456, and AH3422 were included as controls. The experiment was repeated twice with triplicates for each strain. Data show the mean +/− standard deviation, and p < 0.001 by one-way RM ANOVA 5 followed by Student-Newman-Keuls method.

The changes that occur in the transcriptional profile of *S. aureus* upon inactivation or overexpression of MgrA are well established^26^. As a transcriptional regulator, MgrA mediates autolysis in *S. aureus*^29,30^. We tested our MgrA mutants for autolytic activity and found that the Val55Phe mutant behaved similarly to the wild type strain, whereas the Arg84His mutant showed increased sensitivity to treatment with Triton X-100 but was less sensitive than the *mgrA* null mutants (Fig. 5A).

### Mammalian cell toxicity assay

**Figure 6:**
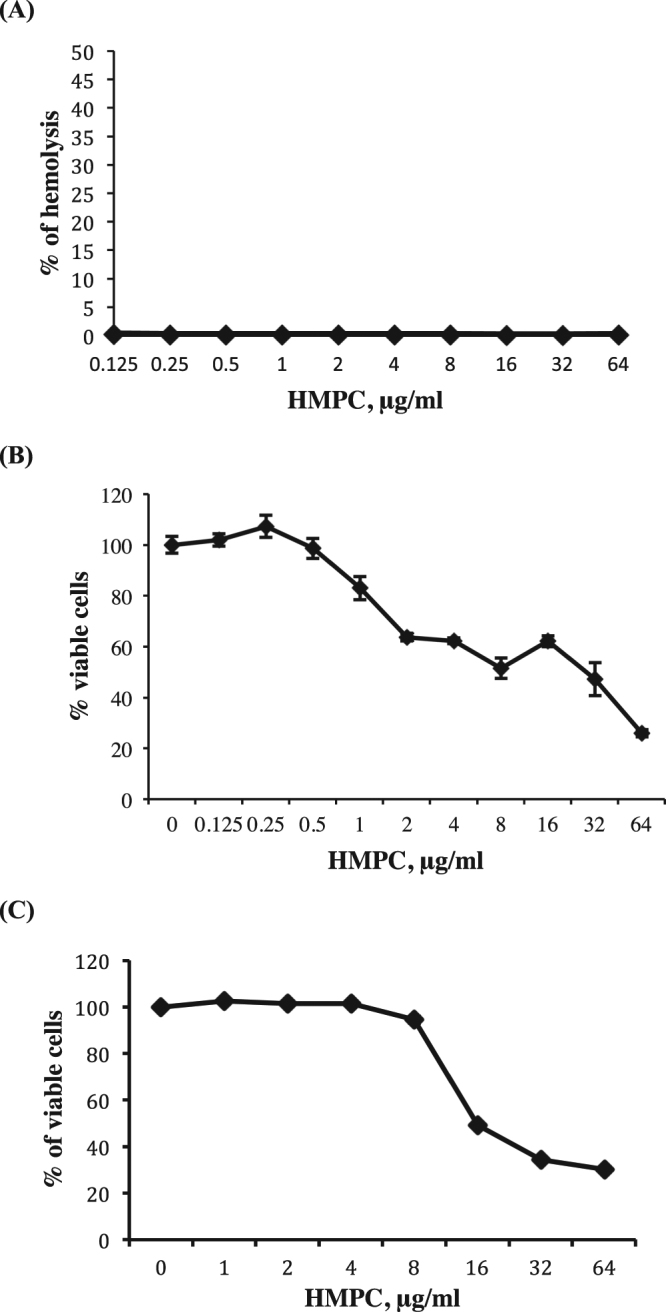
Mammalian cell toxicity assays. (**A**) HMPC hemolysis assay. Red blood cells were diluted to 2% with PBS and then incubated with HMPC over the concentration range 0.125−64 μg/ml. (**B**) Cytotoxicity of HMPC towards HKC-8 cells. HKC-8 cells were treated with increasing concentrations of HMPC for 24 hours before assessing viability using a WST-1 assay. (**C**) Cytotoxicity of HMPC towards HepG2 cells. HepG2 cells were treated with increasing concentrations of HMPC for 24 hours before assessing viability using a WST-1 assay. All experiments were repeated twice in triplicate and data show the mean +/− standard deviation.

As a preliminary assessment of the selectivity of HMPC for bacterial cells over mammalian cells, we performed a hemolysis assay with human red blood cells. HMPC showed no hemolytic activity over the concentration range tested 0.125–64 μg/ml (Fig. 6A). We also assessed the cytotoxicity of HMPC towards HKC-8 kidney cells (Fig. 6B) and found that an approximately 50% reduction in cell viability occurred at concentrations close to its MIC against *S. aureus* MW2. However, HepG2 liver cells were less sensitive to HMPC, with 94% survival at 4 μg/ml and then a decrease to an approximately 50% survival at 16 μg/ml (Fig. 6C). A detailed structure activity analysis of HMPC by replacing or adding modifications to the structural components of the compound and assessing the tradeoffs between toxicity and antimicrobial activity could potentially yield a less toxic analog, which could be used as a prototype lead for development of new antimicrobial agents.

## Discussion

New antibiotics are urgently needed to combat the growing threat posed by multidrug-resistant strains of *S. aureus*. We identified HMPC, a novel anti-staphylococcal compound in the Maybridge 5 screening library, as an antimicrobial agent that inhibited *S. aureus* growth and rescued *C. elegans* from MRSA infection^15^. Here we evaluate the mode of action of HMPC, employ a promoter-*lux* reporter array and whole-genome sequencing of *S. aureus* mutants resistant to HMPC in order to identify possible mechanisms of action, and evaluate the toxicity of HMPC to mammalian cells and *Galleria mellonella* (see Supplementary Fig. S4).

Screening of a *S. aureus* promoter-*lux* array with HMPC revealed a unique transcriptional activation profile for this compound. The compound activated promoter-*lux* clones C, E, F, G, K, L and M, a profile that shows some overlap with DNA-damaging agents (overlapping activation of C, E, F, K, L, M and non-activation of J) and/or DNA replication inhibitors (overlapping activation of C, G, K, L, M and non-activation of J). Because HMPC is a compound with previously unknown antibacterial properties with some overlap to DNA-damaging agents, we tested whether HMPC can directly bind DNA”. However, we did not observe tight binding to calf thymus DNA or genomic DNA from *S. aureus* MW2 in a UV-visible spectra experiment, disc diffusion assay, or electrophoretic mobility shift assay. The promoter-*lux* clones C, E, and to a lesser extent clone F, all of which were activated by HMPC, encode *recA-lux*. We evaluated whether a *recA* mutant strain might show altered susceptibility to HMPC. However, the MIC of HMPC remained unchanged (MIC = 4 μg/ml) against the *S. aureus* JE2 strain and its isogenic mutant lacking a functional RecA protein (NTML, NE805, SAUSA300_1178)^31,32^. The absence of a difference in MIC could further suggest that the SOS response was not due to DNA damage *in vivo*, but rather triggered by a different stressor. It is known, for example, that β-lactam antibiotics trigger SOS responses by compromising the integrity of the bacterial membrane, and SOS induction promotes the appearance of mutations needed for the development of resistance^33^. We tested whether sub-MIC concentrations of HMPC increased the frequency of spontaneous mutations that give rise to rifampicin resistance, but observed no difference between HMPC-treated and untreated bacteria (0.309×10^11^ untreated versus 0.324×10^11^ treated). An Ames test also indicated no potential mutagenicity of HMPC (Table S1). Therefore it appears that despite its *lux*-array profile resembling that of DNA-damaging and SOS-inducing agents, HMPC acts via a distinct mechanism.

Whole-genome sequencing of HMPC-resistant mutants identified two genes that were repeatedly and independently mutated in response to HMPC pressure: the transcriptional regulator *mgrA (MW0648)* and the phosphatidylglycerol lysyltransferase *fmtC/mprF (MW1247)*. MgrA is a global transcription regulator that belongs to the MarR (multiple antibiotic resistance regulator)/SarA (staphylococcal accessory regulator A) family of proteins, which respond to various environmental stressors and play a role in the development of drug resistance^8,34–37^. MgrA affects the expression of approximately 350 genes that control multiple properties of *S. aureus*, such as expression of virulence factors, regulation of autolysis and antibiotic resistance, as well as other global regulatory genes^25,26,29,38–40^. MgrA also regulates the multidrug-resistance (MDR) efflux pumps NorA, NorB and NorC, the non-MDR efflux pump Tet38, and the ABC transporter AbcA in *S. aureus*. These pumps typically provide the first line of bacterial defense against antibiotics until more efficient resistance mechanisms can emerge^41,42^. To test whether drug efflux was playing a role in HMPC resistance, we conducted MIC assays in the presence of the efflux pump inhibitor thioridazine^41,43^. The inhibitor was found to have no effect on *S. aureus* susceptibility to HMPC with MIC value remaining at 4 μg/ml, suggesting that drug efflux is not a major contributor to HMPC resistance.

Clues as to the possible effects of the MgrA mutations that arose in the HMPC-resistant populations could be gathered by assessing their location in the crystal structure of the protein^44^. The Val55Phe and Arg84His variants would clearly introduce steric clashes in the ß-strands near wing 1, a DNA-binding region of the canonical winged-helix domain, which cannot be accommodated without changes to the overall protein structure (Fig. 4). The Pro71Gln mutation maps to the beginning of DNA-recognition helix 4, which would cause extension of the helix and alter the structure of the DNA-binding domain. We predict that the resistance-associated mutations we identified affect the DNA-binding winged-helix domain and thus alter the affinity of MgrA for specific DNA-binding sites, leading to transcriptional changes in the resistant bacteria. The precise nature of these expression changes remains to be explored.

We propose that mutations in MgrA alter the expression of MgrA-regulated genes in response to HMPC, leading to adaptive responses that affect the susceptibility of *S. aureus* and allow other, more stable resistance mutations to appear. This hypothesis was supported by the appearance of secondary mutations in *fmtC (MW1247)* during the course of the resistance selection experiment. FmtC, also referred to as multiple peptide resistance factor (MprF)^45^, catalyzes the formation of lysyl-phosphatidylglycerol during lipid biosynthesis, a modification that increases cell surface positive charge and confers protection against cationic antibiotics^46–50^. It has been suggested that MprF plays a biophysical role in membrane stability and organization, and that this can modify *S. aureus* susceptibility to antibiotics^47,51^. A change in membrane organization that reduces HMPC entry into bacterial cells would explain the increased resistance we observed in the *S. aureus* JE2 *fmtC* mutant strain. In conclusion, neither of the resistance-associated genes we identified is likely to be the cellular target of HMPC. Thus further studies are needed to determine the precise mechanism of action of this compound.

Overall, we report a novel bacteriostatic agent that effectively inhibits the growth of drug-resistant *S. aureus*. While the mechanism of action of HMPC remains unknown, we postulate that it acts on multiple targets within the bacterial cell to induce a stress response, which can be mitigated by transcription changes driven by mutations in MgrA. These changes are followed by a rise in more specific mutations that affect the cellular membrane and possibly reduce entry of HMPC into *S. aureus* cells. Future work will focus on the synthesis of structural analogs that retain anti-staphylococcal activity but are less toxic to mammalian cells.

## Materials and Methods

### Bacterial strains and media

The bacterial strains used in this study are listed in Table 4. Bacterial cultures were grown either in Mueller-Hinton Broth (MHB) (212322, BD Biosciences, MD, USA) or Bacto Tryptic Soy Broth (TSB) (21825, BD Biosciences, MD, USA) liquid medium or on solid agar plates (05040, Agar powder for microbiology, Sigma-Aldrich, MO, USA). The growth of bacterial clones and screening of the *S. aureus* promoter-*lux* array were performed using NYE medium supplemented with 10 μg/ml chloramphenicol (NYEC)^52^.

**Table 4:** Bacterial strains used in this study.

Species	Strain name
*Staphylococcus aureus*, MRSA	MW2 (laboratory collection)
*Staphylococcus aureus*,MRSA	AH843, MW2 (Horswill laboratory)
*Staphylococcus aureus*, MRSA	USA300 JE2 (Department of Pathology and Microbiology, University of Nebraska)
*Staphylococcus aureus* JE2 mutants	Nebraska Transposon Mutant Library (BEI Resources)
*Staphylococcus aureus*,MRSA	AH3456, MW2 *∆mgrA*::tetM (Horswill laboratory)
*Staphylococcus aureus*,MRSA	AH3422, MW2 *∆mgrA* (Horswill laboratory)
*Staphylococcus aureus* MgrA mutant	MW2, R84H mutation in MgrA
*Staphylococcus aureus* MgrA mutant	MW2, V55H mutation in MgrA
*Enterococcus faecium*	E007^60^
*Klebsiella pneumoniae*	WGLW2 (HM-751, BEI Resources)
*Acinetobacter baumannii*	ATCC 17978
*Enterobacter aerogenes* Hormaeche and Edwards	ATCC 13048
*Pseudomonas aeruginosa*	PA14^61^
*Staphylococcus aureus*clinical isolates, MRSA	BFSA25, BFSA30, BFSA31, BFSA32, BFSA33, BFSA48, BFSA49, BFSA50, BFSA51 (Mylonakis laboratory collection)

### Minimum inhibitory concentration, dose response, and antimicrobial killing assays

Propyl 5-hydroxy-3-methyl-1-phenyl-1*H*-pyrazole-4-carbodithioate (HMPC) was purchased from Fisher HealthCare (Thermo Fisher Scientific, TX, USA). The compound was dissolved in 100% DMSO at a concentration of 5 mg/ml. The MIC was determined by a two-fold broth microdilution procedure in 96-well plates in MHB according to standard methods^53^. Dose-response curves were generated by measuring optical density (OD_600_) of the MIC plate after 24 hours of growth using a Synergy plate reader and Gen5 software (Biotek Instruments, VT, USA). For antimicrobial killing assays, log-phase cultures (OD_600_ = 0.3) grown in MHB were treated with 16 μg/ml of HMPC (4xMIC) or 10 μg/ml daptomycin and were incubated at 37 °C with shaking at 220 rpm. Bacterial survival was monitored after 2, 4, 8, and 24 hours by dilutional plating and enumeration of CFU/ml at each time point.

### Growth inhibition of *S. aureus* MW2 by HMPC

An overnight culture of *S. aureus* MW2 was diluted into MHB containing HMPC at varying concentrations and the bacteria were grown at 37 °C for 24 hours. DMSO was used as vehicle control. Bacterial culture aliquots were periodically drawn to measure the optical density at 600 nm (OD_600_) on an Eppendorf BioPhotometer Plus (Eppendorf of North America, NY, USA).

### Screening of *S. aureus* promoter-*lux* reporters

Screening of *S. aureus* promoter-*lux* clones was performed according to the published protocol^21^. In brief, a single colony of each luminescent *S. aureus* clone was re-suspended in sterile water, diluted 1000-fold into 0.7% (w/v) agar and spread evenly across the surface of NYEC plates. Paper discs (232189, BD Biosciences, NJ, USA) containing 2 μg of HMPC were placed onto the plates and incubated at 37 °C for 20 h. Discs containing DMSO were added to plates as controls. Luminescence was detected with an IVIS Lumina III *In Vivo* Imaging System (Perkin Elmer, MA, USA).

### Assessment of HMPC binding to DNA

To assess HMPC binding to eukaryotic DNA, UV-visible spectra were recorded on a Beckman DU 80 spectrophotometer^54^ using a quartz micro cell cuvette. Measurements were obtained for calf thymus DNA in Tris—HCl buffer (pH 7.2, 10 mM) in the presence or absence of HMPC (20 μg/ml) over the wavelength range of 290–400 nm (10 nm increments), as described previously^54^. We also assessed the effect of DNA on the zone of growth inhibition of *S. aureus* produced by HMPC using calf thymus DNA and genomic DNA from *S. aureus* MW2. The experiment with calf thymus DNA was performed as described previously^55^ with minor modifications. Solutions of HMPC (5 μg-25 μg) were mixed with 5 μg calf thymus DNA and incubated for 10 min at RT before being spotted onto discs (232189, BD Biosciences, NJ, USA) and overlaid onto *S. aureus* MW2 lawns growing on MHA medium. The zones of growth inhibition were visually observed and measured with a ruler after incubation at 37 °C for 21 h. Experiments were performed twice.

To assess HMPC binding to prokaryotic DNA, solutions containing 10–25 μg of HMPC were left untreated or were mixed with 5 μg *S. aureus* MW2 genomic DNA. Solutions were then spotted onto filter paper discs and overlaid onto a TSA plate containing MW2 bacteria in a soft agar overlay. After overnight incubation at 37 °C, zones of inhibition were measured with a ruler. Finally, an electrophoretic mobility shift assay (EMSA) was conducted by incubating *S. aureus* MW2 genomic DNA (500 ng) with DMSO alone or with 5 μg HMPC dissolved in DMSO, and then running the mixtures on a 1% agarose gel at 70 volts for 4 hours. The gel was then stained with 0.5 μg/ml ethidium bromide to visualize the DNA bands.

### Determination of the frequency of rifampicin-resistant mutants

An overnight culture of *S. aureus* MW2 was diluted to the McFarland standard 0.5 in TSB medium and grown until mid-log phase. Bacteria were serially diluted 10-fold in TSB medium and aliquots were plated on either rifampicin-containing TSA plates, to determine the number of resistant mutants, or non-selective plates, to determine the number of CFU/ml. The mutation frequency was calculated as the ratio of rifampicin-resistant mutants to total cells in the population, as described previously^56,57^.

### Selection of HMPC-resistant *S. aureus* MW2 mutants

Sequential passaging in liquid medium to develop HMPC-resistant isolates was performed as previously described^24^. For the first round of selection, an overnight culture of *S. aureus* MW2 was diluted to an optical density at 600 nm (OD_600_) of 0.1 in TSB medium supplemented with 1xMIC, 2xMIC, and 4xMIC HMPC (in triplicate). After 24 h of incubation at 37 °C with shaking at 200 rpm, the tube with the highest HMPC concentration that contained visible bacterial growth (OD_600_ > 0.1) was used to dilute the bacterial culture to OD_600_ = 0.1 for the next round of selection, and to determine the concentrations of HMPC to be tested. The MIC of cultures derived from strains grown in the highest HMPC concentration was used to confirm resistance to the compound.

### Whole genome sequencing and variant calling

Genomic DNA from overnight cultures of *S. aureus* MW2 control and resistant strains was isolated using a DNeasy Blood and Tissue kit (Qiagen, CA, USA) using the manufacturer’s protocol. A paired-end sequencing library (2x250 bp) was prepared for each DNA sample using a Nextera XT kit (Illumina, CA, USA). Libraries were pooled and sequenced using an Illumina HiSeq. 2500 (MEEI Ocular Genomics Institute, Boston, MA). Variants were identified using Pilon^58^, by mapping sequencing reads to the closed *S. aureus* MW2 genome (GenBank accession GCA_001019125.1). Unique variants found in the selected strains that were absent in control strains are reported. Additional low-frequency mutations in *mgrA* and *fmtC* were identified using CLC Genomics Workbench version 7.0 (CLC bio, MA, USA).

### Minimum inhibitory concentration of HMPC in the presence of an efflux pump inhibitor

The effect of the efflux pump inhibitor thioridazine on *S. aureus* MW2 susceptibility to HMPC was evaluated using the 2-fold microdilution method in the presence of a sub-inhibitory concentrations of thioridazine (0.5 μg/ml), as previously described^41^.

### Triton X-100 – induced autolysis assay under static conditions

Overnight cultures of *S. aureus* strains were diluted to an optical density at 600 nm (OD_600_) of 0.1 in TSB with 0.05% Triton X-100. Cells were incubated in 96-well plates at 37 °C without shaking, and optical densities were recorded hourly for 8 hours.

### Mammalian cell cytotoxicity assay

Growth media and other cell culture reagents were obtained from Thermo Fisher Scientific. HKC-8 immortalized human renal proximal tubular epithelial cells were maintained in Dulbecco’s Modified Eagle Medium/Nutrient F-12 Ham (DMEM/F12) supplemented with 10% FBS. The HepG2 liver cells were from ATCC (HB-8065) and cells were grown in Eagle’s Minimum Essential Medium (EMEM) supplemented with 10% FBS. HKC-8 and HepG2 cells were cultured in 96 - well tissue culture plates at a density of 10000 cells/well, followed by the addition of HMPC in various concentrations or control medium (DMEM/F12 or EMEM with 0.01% DMSO). The cells were subsequently incubated for 24 hours prior to the application of Cell Proliferation Reagent WST-1 (WST-1 reagent Roche, IN, USA), according to the manufacturer’s instructions. Each concentration of HMPC was tested in triplicates and the experiment was repeated twice.

### Hemolysis assay

Human erythrocytes were purchased from Rockland Immunochemicals (Human red blood cells 10% washed pooled cells, R407-0050). The assay was performed as described^59^ in triplicates and repeated twice.

## Electronic supplementary material

Supplementary Information
