# Supplementary material for: Propyl-5-hydroxy-3-methyl-1-phenyl-1H-pyrazole-4-carbodithioate (HMPC): a new bacteriostatic agent against methicillin—resistant Staphylococcus aureus
Source: Sci Rep. 2018 May 4;8:7062. doi: 10.1038/s41598-018-25571-w (PMC5935714; doi:10.1038/s41598-018-25571-w)
Supplement: Supplementary file 1 — Supplementary Information [file 41598_2018_25571_MOESM1_ESM.pdf]

Propyl-5-hydroxy-3-methyl-1-phenyl-1*H*-pyrazole-4-carbodithioate (HMPC): a new bacteriostatic agent against methicillin—resistant *Staphylococcus aureus*

Tatiana Johnston[1], Daria Van Tyne[2,3], Roy F. Chen[1], Nicolas L. Fawzi[4], Bumsup Kwon[5], Michael J. Kelso[6], Michael S. Gilmore[2,3] & Eleftherios Mylonakis ∗,[1]

[1]Department of Infectious Disease, Rhode Island Hospital, Alpert Medical School of Brown University, Providence, Rhode Island, USA, [2]Department of Ophthalmology, Harvard Medical School, Massachusetts Eye and Ear Infirmary, Boston, Massachusetts, USA, [3]Department of Microbiology and Immunobiology, Harvard Medical School, Boston, Massachusetts, USA, [4]Department of Molecular Pharmacology, Physiology, and Biotechnology, Brown University, Providence, Rhode Island, USA, [5]Department of Neurology, Rhode Island Hospital, Warren Alpert Medical School of Brown University, Providence, RI 02903, USA, [6]School of Chemistry and Illawarra Health and Medical Research Institute, University of Wollongong, NSW 2522, Australia. Correspondence and requests for materials should be addressed to E.M. (email: emylonakis@lifespan.org)

Supplemental Methods

Ames test. To assess the potential mutagenic potency of HMPC the Ames test was performed according to the provided by manufacture protocol (Fisher Scientific, NH, USA, NC9159443). The range of concentrations of HMPC from 4 μg to 64 μg was tested and colony counts compared to a negative control (DMSO) and the positive control 4-NOPD (4-nitro-o-phenylenediamine; carcinogen).

***Galleria mellonella* toxicity studies.** *Galleria mellonella* larvae were purchased from Vanderhost Wholesale (OH, USA) and stored in the dark at room temperature upon arrival until used. The *G. mellonella* manipulation techniques were performed according previously published protocol[1]. In brief, larvae weighing 230-250 mg were selected for experiments. Each group included 10 larvae and experiments were repeated twice using larvae from independent batches. There were two negative control groups in each experiment, the first group underwent no manipulation and the second was injected with PBS only for control of the impact of physical trauma on larvae. Larvae were injected in the last left proleg using 10 μl Hamilton syringe (20779, Sigma Aldrich, MO, USA) with HMPC at concentrations of 4, 8 and 10 mg/kg. Later the larvae in Petri dishes were placed into 37 °C incubator and their survival was monitored each 24h for 5 consecutive days. Larvae were scored for survival by observing for movement after gentle touch.

Supplemental Tables

Table S1. Ames test to assess the potential mutagenicity of HMPC.

Name of chemical	Average number of colonies[a]	+/- STDEV
4-NOPD	174	6
DMSO alone	55	4
4 μg HMPC	41	4
8 μg HMPC	62	8
16 μg HMPC	49	2
32 μg HMPC	42	2
64 μg HMPC	39	3

[a]The test was performed in triplicate and the numbers of colonies were rounded to the next whole number.

Supplemental Figures

Figure S1

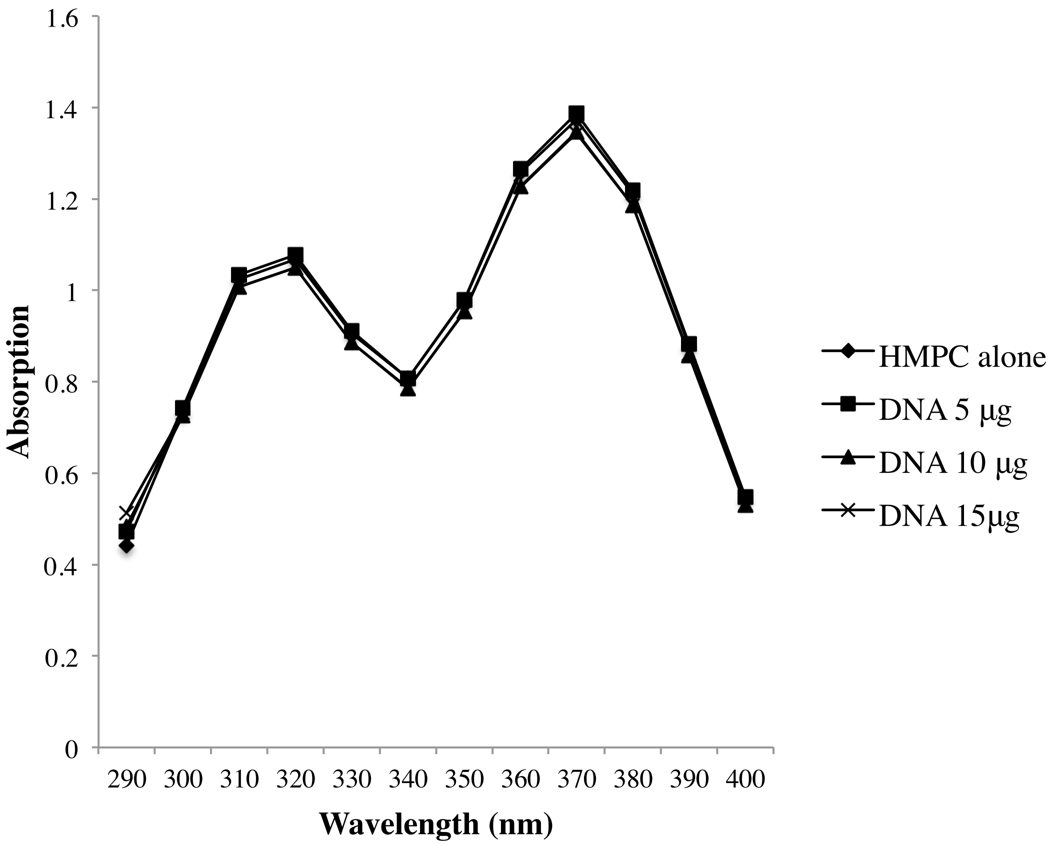

Figure S1. UV-visible absorption spectra of HMPC alone and in the presence of increasing concentration of DNA in Tris-HCl buffer (pH7.2). No significant hyperchromism or hyperchromism was observed with increasing concentration of DNA confirming no interaction of HMPC and DNA.

Figure S2

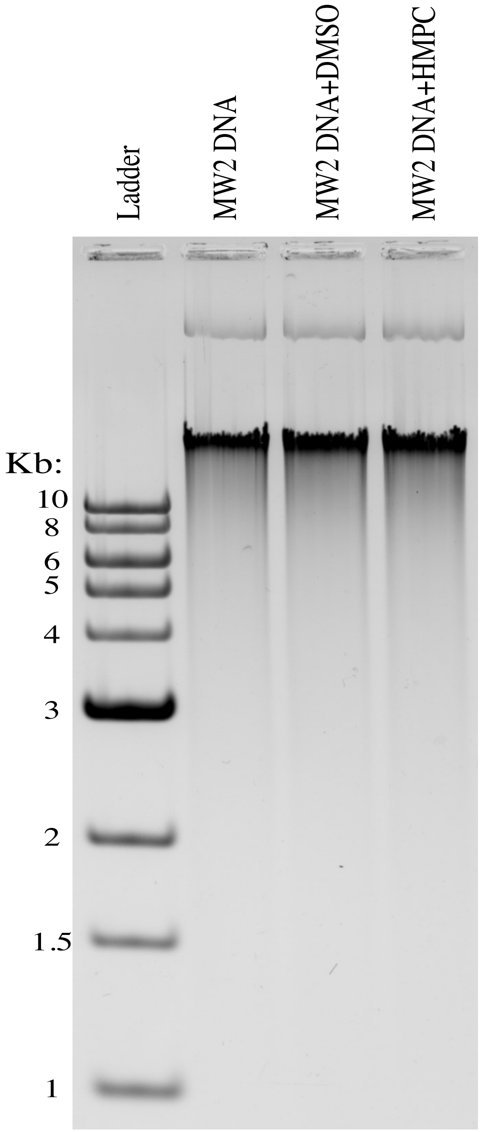

Figure S2. Electrophoretic mobility shift assay (EMSA) to detect binding between HMPC and *S. aureus* MW2 genomic DNA. DNA (500ng) was incubated with DMSO alone, or with 5μg HMPC dissolved in DMSO before being run on a 1% agarose gel at 70 volts for 4 hours. The gel was then stained by incubating in 0.5μg/ml ethidium bromide. The contrast was not adjusted and the image was not processed or manipulated other than to crop the blank areas of the gel.

Figure S3

(A)

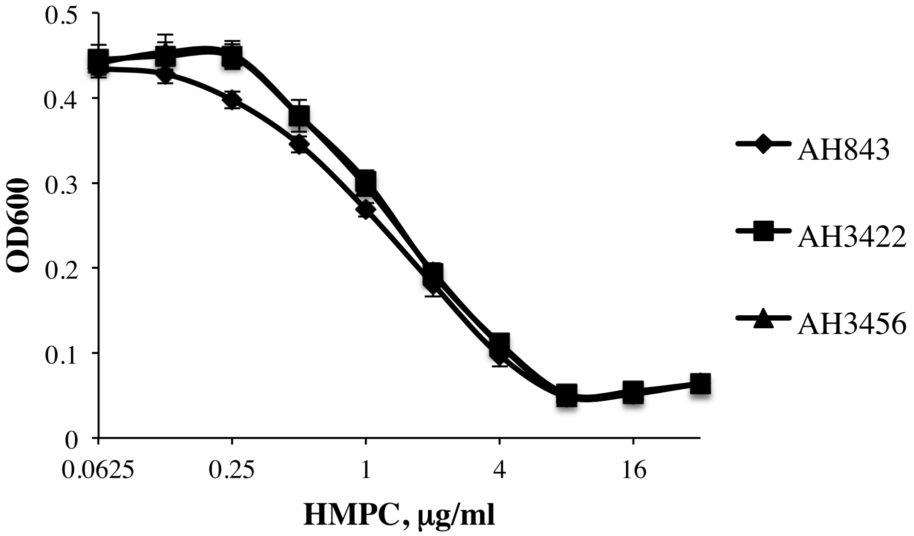

(B)

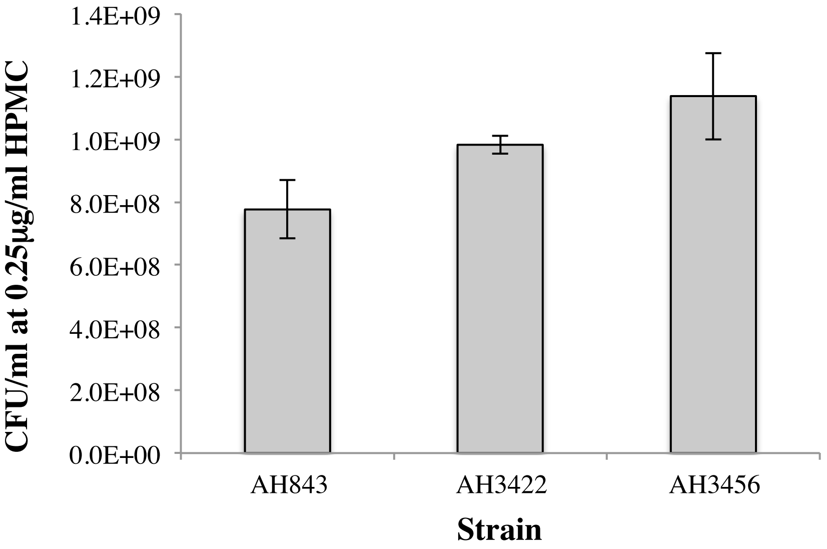

Figure S3. Mutants that lack a functional *mgrA* are more tolerant of sub-MIC levels of HPMC. (**A**) Dose-response curves for the AH843 wild-type strain and two mutant strains that have a disrupted *mgrA* locus (AH3422 and AH3456). All strains were inoculated at the same cell density into MH broth containing varying concentrations of HMPC, and OD_{600} was measured after 24 hours of growth at 37 °C. (**B**) Cell viability after growth in

0.25 μg/ml HMPC. Bacterial strains grown in the presence of 0.25 μg/ml HMPC for 24 hours were serially diluted and plated onto TSA plates with no drug and were grown overnight at 37 °C in order to enumerate CFU/ml. In both panels, mean +/- standard deviation of at least three replicates is shown. p-values for comparison of *mgrA* mutants to AH843 wild type strains in panel B using a two-tailed t-test are p=0.02 for AH3422 and p=0.01 for AH3456.

Figure S4.

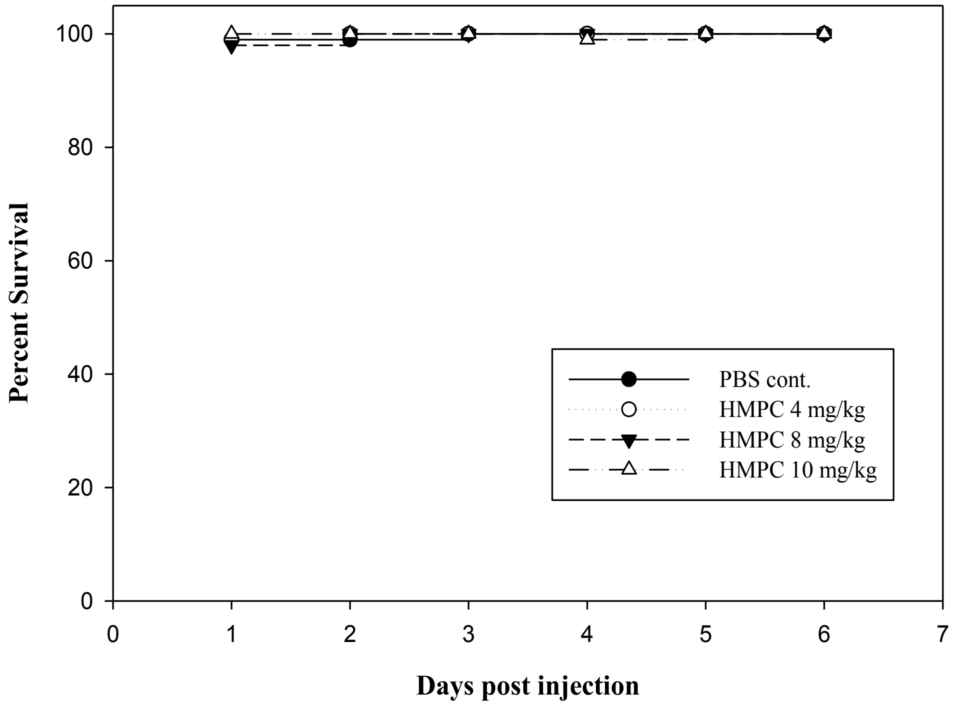

Figure S4. *Galleria mellonella* toxicity experiment. Graph represents combined data from two independent experiments (each group had n=10 larvae, no larvae death observed in no injection control group). There was no difference in larvae survival between the groups (one way ANOVA4, P=0.000).

References

1. Desbois, A. P. & Coote, P. J. Wax moth larva (Galleria mellonella): an in vivo model for assessing the efficacy of antistaphylococcal agents. *J. Antimicrob. Chemother.* **66**, 1785-1790, doi:10.1093/jac/dkr198 (2011).
